# Knowledge and Practices during the COVID-19 Outbreak in the Middle East: A Cross-Sectional Study

**DOI:** 10.3390/ijerph18094699

**Published:** 2021-04-28

**Authors:** Abdallah Y. Naser, Eman Zmaily Dahmash, Zahra Khalil Alsairafi, Hassan Alwafi, Hamad Alyami, Zahraa Jalal, Ahmed M. Al Rajeh, Vibhu Paudyal, Yosra J. Alhartani, Fawaz Mohammad Turkistani, Fadi Fouad Hassanin

**Affiliations:** 1 Department of Applied Pharmaceutical Sciences and Clinical Pharmacy, Isra University, Amman 11622, Jordan; eman.zmaily@iu.edu.jo (E.Z.D.); yosra.alharatni@hotmail.com (Y.J.A.); 2 Department of Pharmacy Practice, Kuwait University, Kuwait City 12037, Kuwait; zahra.alsairafi@hsc.edu.kw; 3 Department of Pharmacology and Toxicology, Umm al-Qura University, Mecca 21514, Saudi Arabia; hassan.alwafi.16@ucl.ac.uk; 4 Department of Pharmaceutics, Najran University, Najran 1988, Saudi Arabia; hsalmukalas@nu.edu.sa; 5 School of Pharmacy, University of Birmingham, Edgbaston, Birmingham B15 2TT, UK; z.jalal@bham.ac.uk (Z.J.); v.paudyal@bham.ac.uk (V.P.); 6 Department of Respiratory Care, King Faisal University, Al Hofuf 31982, Saudi Arabia; amalrajeh@kfu.edu.sa; 7 Alnoor Hospital, Mecca 21514, Saudi Arabia; fm_turkistani@hotmail.com; 8 Ophthalmology Department, College of Medicine, Jeddah University, Jeddah 2749, Saudi Arabia; ffhassanin@gmail.com

**Keywords:** COVID-19, Jordan, knowledge, Kuwait, practices, Saudi Arabia

## Abstract

*Objectives:* This study aimed to assess the knowledge and practices of the general public in the Middle Eastern countries during the COVID-19 pandemic. *Methods:* A cross-sectional study using an online survey was conducted between the 19th of March and the 6th of April 2020 in three Middle Eastern countries (Jordan, Saudi Arabia, and Kuwait) to explore the knowledge and practices of the Middle Eastern population regarding COVID-19. A previously developed questionnaire was adapted and used for this study. Multiple linear regression analysis was used to identify predictors of COVID-19 knowledge. *Results:* A total of 1208 participants (members of the public) participated from the three countries (Jordan = 389, Saudi Arabia = 433, and Kuwait = 386). The majority of participants (*n* = 810, 67.2%) were females aged 30 to 49 years (*n* = 501, 41.5%). Participants had moderate overall COVID-19 knowledge, with a mean (SD) score of 7.93 (±1.72) out of 12 (66.1%). Participants had better knowledge about disease prevention and control (83.0%), whereas the lowest sub-scale scores were for questions about disease transmission routes (43.3%). High education level was an important predictor of greater COVID-19 knowledge scores (*p* < 0.01). *Conclusions:* Further public education is needed to address the relatively low level of education regarding the transmission of COVID-19 in the Middle Eastern countries. Policymakers are recommended to develop informative COVID-19 related campaigns that specifically target young people (university students), unemployed individuals, and those with lower levels of education.

## 1. Introduction

Coronavirus disease 2019 (abbreviated as “COVID-19”), an infectious disease with unknown treatment characterised by acute pneumonia, was first recognised in Wuhan, China in December 2019. It is predominantly characterised by fever, fatigue, and a dry cough. A recent study among more than 370,000 patients with confirmed COVID-19 reported that the predicted probability of hospitalisation was 38.4% (37.6–39.2) for patients with diabetes only and 42.9% (42.2–43.7) for patients with diabetes and one or more comorbidities (obesity, hypertension, cardiovascular disease, and chronic kidney disease). On 12 March 2020, the World Health Organisation (WHO) declared that the COVID-19 outbreak is a pandemic [1]. The pandemic has lingered and has had a hefty toll on healthcare professionals, families, communities, and the whole world. In response to the pandemic, Jordan, Saudi Arabia, and neighbouring Kuwait took drastic measures early on in a bid to contain the disease, including halting air travel, imposing curfews, and quarantining and testing thousands of individuals [2,3].

Once a new pandemic starts, the focus of research and action within the medical and public health communities is chiefly and rightly directed towards the identification of the cause, clinical presentation, diagnosis, and treatment [4]. Few studies address the epidemiology of the disease, the effectiveness of preventive measures, and the population’s psycho-behavioural directions [5,6,7,8]. Nevertheless, addressing public health preventive measures deserves equal attention. Although several ongoing clinical trials are assessing potential vaccines and treatments for COVID-19, currently, there is no specific treatment that can fully combat the virus within medical practice. Therefore, public health measures are of crucial value. Such measures mandate the public to be aware of transmission means and have safe preventive practices [9]. In this regard, people have concerns about the safety measures to be taken to protect themselves and their families from being infected. Generally, the spread of any infectious disease is associated with a high level of fear among the population [10]. A particular concern in this regard is the spread of misinformation about COVID-19 on social media sites [11]. Universal data collection and analysis of pandemic control contains little information about the knowledge and practices of the population regarding the disease and its significance [12,13]. To guarantee the best control over the transmission of the disease, the community’s adherence to important control measures is vital, which is affected to large extent by their knowledge about COVID-19 and their preventative practices [12,13]. Thus, there is an urgent need to understand what people know about COVID-19, and which misperceptions they hold about this condition, to help healthcare authorities and the media design effective information campaigns, to know which groups of people should be targeted, and to help to control the spread of the virus among the population. Therefore, this study aims to assess the Middle Eastern population’s knowledge about COVID-19 and their practices during the disease’s rapid rise period. 

## 2. Methods

### 2.1. Study Design and Study Population

A cross-sectional study using an online survey was conducted between the 19th of March and the 6 April 2020 in three Middle Eastern countries (Jordan, Saudi Arabia, and Kuwait) to explore the knowledge and practices of the Middle Eastern population concerning COVID-19. 

### 2.2. Sampling Strategy

A convenience sample of eligible participants was invited to participate in the study from the three countries through social media platforms. All participants voluntarily participated in the study, and consent was gained when filling in the questionnaire. The study aim and objectives were clearly explained at the beginning of the survey. The inclusion criterion was participants aged 18 years who were living in Jordan, Saudi Arabia, or Kuwait. Participants were excluded if they were (a) below 18 years of age or (b) unable to understand the Arabic language.

### 2.3. Questionnaire Tool

A previously developed questionnaire by Zhong et al. was adapted and used in this study [14]. The original questionnaire was developed based on the National Health Commission of the People’s Republic of China’s guidelines for clinical and community management of COVID-19 [15,16]. The original questionnaire had 12 questions (true/false questions): four regarding clinical presentations (K1–K4), three regarding transmission routes (K5–K7), and five regarding prevention and control (K8–K12) of COVID-19. These questions were answered on a true/false basis with an additional “I don’t know” option. A correct answer was given a score of one point, and any false or unknown answer was given a score of zero points. The total knowledge score could range from 0 to 12, with a higher score indicating a better knowledge about COVID-19. Cronbach’s alpha coefficient of the knowledge questionnaire was 0.7 in our sample, showing acceptable internal consistency. Respondents’ practices were assessed using two behaviour questions, which asked the participants about whether they have gone to any crowded places, and wearing a mask when going out in recent days. In addition, the following information was collected about the participants’ demographics: age, gender, marital status, education level, income, and employment status.

### 2.4. Sample Size 

The researchers chose to apply a standard deviation of 0.5 and a margin of error of 5% in this study. To power the study, the minimum targeted sample size was 385 participants from each study population to have a 95% chance to detect a difference.

### 2.5. Statistical Analysis

Descriptive statistics were used to describe participants’ demographic characteristics. Continuous data were reported as mean ± SD. Categorical data were reported as percentages (frequencies). Independent samples t-test/one-way analysis of variance (ANOVA) were used to compare the mean knowledge scores between different demographic groups. Participants’ scores were interpreted as a continuous scale based on the scale midpoint, where scores above the midpoint identified stronger COVID-19 knowledge. The mean score was expressed in the percentage out of 100% to facilitate the comparison between different sub-scales. Multivariable linear regression analysis was used to identify predictors of knowledge score. A two-sided *p* < 0.05 was considered statistically significant. The statistical analyses were carried out using Statistical Package for Social Science (SPSS) software (version 25) (IBM, Chicago, IL, United States)).

## 3. Results

### 3.1. Participants’ Characteristics

A total of 1208 participants took part in this study (Jordan = 389, Saudi Arabia = 433, and Kuwait = 386). Table 1 details the baseline characteristics of the participants in the three countries.

The majority of participants (*n* = 810, 67.2%) were females, aged 30 to 49 years (*n* = 501, 41.5%), married (*n* = 685, 56.8%), had a bachelor degree (*n* = 723, 60.4%), employed (*n* = 568, 47.1%), and with an income of $2113 and above (*n* = 497, 44.6%). Around 15.3% (*n* = 183) of the participants reported that they had been in crowded places recently. Around 50.1% (*n* = 605) of the participants reported that they had worn a mask when leaving their homes.

Table 2 below shows the mean scores (and score out of 100%) for the COVID-19 knowledge questionnaire per subscale. The overall COVID-19 knowledge score was 66.1%. Participants had better knowledge about disease prevention and control with 83.0%, whereas the lowest sub-scale scores were for questions about disease transmission routes (43.3%).

Table 3 below details the correct answer rates of the 12 questions on the COVID-19 knowledge questionnaire. Participants had the highest rates of correct answers for questions related to the necessity of isolation and some procedures related to prevention and control of the infection (K10–K12). On the other hand, the lowest rates of correct answers were for questions related to the susceptibility of children and young adults to COVID-19, transferability of the infection from asymptomatic patients, and eating habits related to COVID-19 transmission (K5, K6, and K9).

### 3.2. Participants’ Demographics and COVID-19 Knowledge

Table 4 below presents participants’ demographics data and their COVID-19 knowledge scores. Participants’ knowledge scores significantly differed by country, age, marital status, education level, and whether they wear a mask upon leaving home or not (*p <* 0.05).

Multiple linear regression analysis showed that individuals aged 50 years and above had a greater knowledge score (*p <* 0.05). On the other hand, individuals who are from Saudi Arabia or Kuwait, divorced individuals, and university students had a lower knowledge score (*p <* 0.05), as shown in Table 5.

## 4. Discussion

To the best of our knowledge, this is the first study that assesses the knowledge and practices regarding COVID-19 among the Arabic-speaking Middle Eastern countries. The results revealed that participants are still embracing misconceptions about COVID-19, resulting in inadequate practices of protective measures against COVID-19 infection. However, some of the respondents in this study who did not adhere to protective measures could be working in sectors that necessitate them being in crowded places, such as port employees or workers in the healthcare sector. The findings of this study revealed that the overall knowledge score among the three countries was around 66.1% (score 7.93 out of 12), with the highest being among Jordanians with a mean score of (8.44 (±1.46); 70.3%), which indicates an average level of knowledge about the pandemic. 

Among the three countries, the participants achieved the lowest knowledge score in the “transmission routes” sub-scale, with a score of 43.3%. The highest knowledge score was recorded on the “preventive and control measures” sub-scale, with a score of 83.0%. Overall, the total knowledge score (66.1%) was moderate, which was unexpected, as this epidemiological survey was conducted during the advanced stage of the pandemic. Although the low percentage of 15.3% (183/1194) with regard to the reported practices of attending crowded places was encouraging, only 50.1% (605/1195) of participants declared that they wore masks when leaving home. The World Health Organisation (WHO) in February 2020 indicated that there is a priority for research to focus on actions that can save lives, which included *“optimal use of protective equipment and other infection prevention and control measures in health care and community settings. It is critical to protect health care workers and the community from transmission and create a safe working environment”* [13]. The need to avoid crowds and wear masks upon leaving the house are key practices and top priorities of the WHO. A similar study conducted by Zhong et al. in China using an online survey [14] showed that the knowledge score among Chinese residents was 90.0% and citizens were compliant with protective measures, where 96.4% avoided crowded places and 98.0% wore masks upon departing their homes during the COVID-19 outbreak [14]. These results undoubtedly revealed that there were significant gaps in the public knowledge about COVID-19 within the Arabic-speaking Middle Eastern countries and suggest the need for improving individuals’ COVID-19 knowledge via health education, which may improve their practices in preventing the transmission of COVID-19 [14].

Although the pandemic is severe and the news reports on this public health emergency are disseminated through various channels of information and via Ministry of Health hotlines, a lot of people learn about this infectious disease from invalid social media resources such as WhatsApp, Instagram, Twitter, etc. This has also been mentioned in an online survey study by Gelsetzer et al. [17], where it has been noticed that a substantial proportion of people in the United States (US) and the United Kingdom (UK) believe in the “myth busters” who continue to circulate inaccurate information about COVID-19. Such information includes rinsing the nose with saline solution, using a hand dryer, taking antibiotics, and gargling with mouthwash as effective preventive measures against COVID-19, and that receiving a letter or package from China may pose a risk of COVID-19 infection [18]. Other unreliable beliefs of the general public in both the US and the UK about COVID-19 are that it is a fatal disease and that children are at higher risk of death from it. Another fact that people did not believe was that medical masks are highly effective in preventing COVID-19 infection [18]. However, people in the Middle East region have reported both similar and different beliefs. For example, in the current study, most participants (66.9%) reported that children and younger adults do not have to take preventive measures against COVID-19. Thus, in contrast to people in the US and UK, people in the Middle East believe that children and younger adults are at lower risk of COVID-19 infection [18]. On the other hand, most people (57.0%) in the Middle East shared similar beliefs regarding wearing medical masks to prevent them from being infected with COVID-19. Regarding the fatality risk of COVID-19, Middle East participants reported conflicting beliefs to people in the US and UK, as the majority (89.0%) believe that although there is no effective treatment, supportive measurements and symptom alleviation can cure the infection [18]. 

Unfortunately, the present study showed that 15.3% of participants still go to crowded places and around 50.0% do not wear masks when leaving their homes. However, not wearing a mask when leaving home could be related to the fact that most of the participants (84.7%) reported being cautious and that they avoided crowded places. This strict preventive practice from the participants could be primarily attributed to the very strict prevention and control measures that were implemented by local governments such as traffic limits throughout the countries and the shutdown of shopping malls and work in all sectors except the health sector. Secondly, this could be due to the population’s fear of the virus and its high infectivity. In China, although the study by Zhong et al. was conducted during the very early stage of the pandemic, people achieved a correct rate of 90.0% on the COVID-19 knowledge questionnaire [14]. In addition, the population’s adherence to the preventive and control measures was higher than in the Middle East, where almost all people stayed at home during the virus outbreak period and wore masks when leaving their homes. This could be attributed to the more serious situation of the COVID-19 pandemic in China, where higher rates of death were recorded at that particular time, resulting in stricter adherence to preventive measures. 

Further analysis of the findings was directed towards determining demographic factors associated with knowledge gaps among participants. Such findings will be valuable for public-health policymakers and healthcare professionals to recognise target populations for health education activities concerning the COVID-19 outbreak. This is critical, as the countries involved at the time of this study were still at the beginning of the outbreak, and governments took extreme measures to contain the pandemic. However, such measures, which include imposing a curfew, are virtually useless unless accompanied by individuals taking responsibility for practising preventative measures [2]. In this study, as well as in previous studies in China, the US, and the UK, an association has been noticed between particular demographic characteristics, knowledge, beliefs, and practices towards COVID-19 [14,17]. For example, in China, an association between non-adherence to preventative and control measures was linked to being male, being a student, having a marital status of “other”, residing in other parts of China, and poor COVID-19 knowledge [14]. These findings were supported by previous studies regarding age and gender patterns of risk-taking behaviours [19,20]. In the US and UK, Geldsetzer et al. reported lower fatality rates due to COVID-19 infection among individuals of East-Asian ethnicity and children [17]. In the current study, there was a significant difference between knowledge scores in the three countries included in the analyses, and the highest knowledge score among the participants has been found in Jordan. Additionally, both males and females showed similar levels of knowledge about COVID-19. 

Higher COVID-19 knowledge scores were found to be significantly associated with age, which is in line with the study conducted in China during the COVID-19 pandemic [14]. Older individuals above the age of 50 years showed a significant increase in knowledge scores than younger individuals. This could suggest that the contents and forms of health-related information about the pandemic could not be understandable and acceptable to young adults and less educated individuals [21]. Our findings are consistent with several previously published studies that linked age and the level of education with knowledge and awareness about outbreaks [22,23]. 

Furthermore, several studies reported that the lack of knowledge contributed to the emergence and spread of the outbreak. Therefore, providing targeted health education programmes to raise awareness is vital. Such programmes need to be tailored to young individuals and particularly those with lower education levels [23,24,25]. 

Furthermore, a significant negative effect on the knowledge score was employment status, specifically among students (*p* < 0.05). The results are aligned with the finding that a lower knowledge score was observed among younger people (below 50 years). Such findings are alarming, as the lack of knowledge about COVID-19 is correlated with a higher prevalence rate of the infection [14,26]. Therefore, there is a need to improve students’ knowledge about COVID-19 through health education, which may also result in improvements in their practices towards COVID-19. Such results could also support the decision made by many countries, including Jordan, Kuwait, and Saudi Arabia, to temporarily close all educational institutions to contain the spread of COVID-19. Globally, the action involved 188 countries, affecting around 1.5 billion learners. The mode of teaching moved into emergency remote learning [26,27]. Yet, although the closure of schools and educational facilities is a good way to prevent young adults from mixing with others, it will not prevent exposure, even with curfew laws. Young adults will always go out and risk exposure to the infection as the level of knowledge is low. A recent study on COVID-19 concluded that educational institutions’ closures alone would preclude only 2–4% of deaths, which is a lot less than other social distancing interventions. The integration of additional social distancing strategies along with educational institutions’ closures needs to be considered [28]. Furthermore, the results of our study pertinent to knowledge scores stratification according to demographics are in line with studies on COVID-19 in China and severe acute respiratory syndrome (SARS) outbreaks, among others [14,29]. These findings also propose that health education strategies would be more effective if they are designed to target certain demographic groups, such as university students.

Good knowledge is vital to enable individuals to have better practices in pandemics and outbreaks. A study on SARS demonstrated that a higher knowledge score was found to be connected with improved adherence to precautionary practices [15,30,31]. Of interest, the income level did not have a significant association with the knowledge score in our cohort, showing that high income does not improve knowledge and practices. This confirms the findings in a previous study on the influenza pandemic, where there was no association between knowledge and income [30]. The current study showed that knowledge scores differed significantly across marital status categories (*p* < 0.01); divorced participants had a significantly negative association with the COVID-19 knowledge score (*p* < 0.05). Our findings were in line with the results obtained from the study on COVID-19 in China, which revealed a statistically significant difference in knowledge score according to marital status; however, they found that the category denoted as “other” (including divorced and widowed, separated, remarried) showed the highest score in knowledge (11.0 out of 12.0) (91.7%), *p* <0.001) [14]. This difference could be justified by two elements. Firstly, different countries might have different cultural dimensions, and hence, results could not be compared. Secondly, the group used in the Chinese study included all divorced, separated, widowed, and remarried individuals, and hence, we could not build a good correlation [14].

Overall, knowledge scores within our cohort were significantly associated with a higher likelihood of risky practices towards the COVID-19 pandemic. Such findings indicate the presence of a knowledge gap and the need to improve individuals’ COVID-19 knowledge via extensive health education strategies. Such strategies are to be tailored and targeted towards specific demographic groups, particularly university students, individuals with low education, and divorced individuals.

### Strengths and Limitations

To the best of our knowledge, this is the first study in the Arabic-speaking Middle Eastern countries that investigated the public’s knowledge and practices during the COVID-19 pandemic. A strength of the study is that a large sample of participants was recruited during this critical period—the actual COVID-19 outbreak—and hence replies reflect the actual status. The participants were from three countries, namely Jordan, Saudi Arabia, and Kuwait, which increases the generalisability of these findings. Additionally, the use of a previously used assessment tool that allowed comparison with other populations was another strength of the study. The study design itself, a cross-sectional survey design, limited our ability to identify causality between study variables. There are limited studies that have assessed knowledge and practices of individuals during the COVID-19 pandemic worldwide and in the Middle East specifically, which limited our ability to compare our findings with Arabic-speaking countries of a similar socioeconomic level and culture. In this study, we employed a quantitative methodology with pre-set responses, which might not have allowed participants’ views to provide varied but useful qualitative information. In addition, we used an online survey for data collection and, therefore, vulnerable populations within the three countries under the COVID-19 pandemic (who may not have had access or were not actively using social media) could not be reached, and we may have missed some of the targeted population (selection bias). The analyses in this study were not preregistered, which could affect the interpretation of the statistical analysis. Finally, we were not able to estimate the response rate for our questionnaire study.

## 5. Conclusions

The findings of this study suggest that general members of the public in the Middle Eastern countries demonstrated a relatively low level of knowledge about COVID-19, particularly regarding its transmission routes. The majority of the population showed appropriate practice by avoiding crowded places and staying home during the rapid rise period of the COVID-19 outbreak. However, not wearing a mask when leaving home was predominant. As good COVID-19 knowledge is associated with optimistic attitudes and appropriate practices towards COVID-19, policymakers should develop targeted information campaigns provided to specific populations such as university students, unemployed individuals, and those with lower levels of education. This information could be conveyed by clinicians to their patients and through news coverage supplied by the media and social media platforms.

## Figures and Tables

**Table 1:** Participants’ characteristics from each country.

Demographics	Overall (*n* = 1208)	Jordan (*n* = 389)	Saudi Arabia (*n* = 433)	Kuwait (*n* = 386)
**Gender** (*n* = 1205) No. (%)				
Female	810 (67.2)	280 (72.0)	324 (75.0)	206 (53.6)
**Age** (*n* = 1206) No. (%)				
18–29 years	424 (35.2)	237 (61.1)	131 (30.3)	56 (14.5)
30–49 years	501 (41.5)	112 (28.9)	189 (43.6)	200 (51.9)
50 years and above	281 (23.3)	39 (10.1)	113 (26.1)	129 (33.5)
**Marital status** (*n* = 1207) No. (%)				
Single	429 (35.5)	213 (54.8)	148 (34.2)	68 (17.7)
Married	685 (56.8)	161 (41.4)	241 (55.7)	283 (73.5)
Divorced	57 (4.7)	5 (1.3)	31 (7.2)	21 (5.5)
Widowed	36 (3.0)	10 (2.6)	13 (3.0)	13 (3.4)
**Education level** (*n* = 1198) No. (%)				
Completed secondary grade or lower	274 (22.9)	48 (12.5)	105 (24.3)	121 (31.7)
Completed bachelor degree	723 (60.4)	283 (73.7)	244 (56.5)	196 (51.3)
Completed higher education	201 (16.7)	53 (13.8)	83 (19.2)	65 (17.0)
**Employment status** (*n* = 1206) No. (%)				
Retired	158 (13.1)	13 (3.3)	63 (14.5)	82 (21.4)
Unemployed	278 (23.1)	113 (29.0)	116 (26.8)	49 (12.8)
Employed	568 (47.1)	147 (37.8)	185 (42.7)	236 (61.5)
University students	202 (16.7)	116 (29.8)	69 (15.9)	17 (4.4)
**Income categories** (*n* = 1115) No. (%)				
705 $ or lower	368 (33.0)	200 (58.3)	130 (32.5)	38 (10.2)
705 $–1408 $	175 (15.7)	96 (28.0)	62 (15.5)	17 (4.6)
1408 $–2113 $	75 (6.7)	15 (4.4)	26 (6.5)	34 (9.1)
2113$ and above	497 (44.6)	32 (9.3)	182 (45.5)	283 (76.1)
**In recent days, have you gone to any crowded place?** (*n* = 1194) No.(%)				
Yes	183 (15.3)	85 (21.9)	38 (8.9)	60 (16.0)
**In recent days, have you worn a mask when leaving home?** (*n* = 1195) No.(%)				
Yes	605 (50.1)	204 (52.6)	191 (44.3)	210 (55.9)

**Table 2:** Participants’ mean scores for COVID-19 knowledge per subscale.

Questions Category	Number of Items	Range	Mean (± SD)	Participants Score Out of 100%
Clinical presentations	4	0–4	3.17 (0.98)	79.2
Transmission routes	3	0–3	1.30 (0.64)	43.3
Prevention and control	5	0–5	4.15 (1.04)	83.0
**Total Knowledge Scale**	12	0–12	7.93 (1.72)	66.1

**Table 3:** The correct answer rates of the COVID-19 knowledge questionnaire.

Questions	%
K11. Isolation and treatment of people who are infected with the COVID-19 virus are effective ways to reduce the spread of the virus.	98.4
K10. To prevent the infection by COVID-19, individuals should avoid going to crowded places such as train stations and avoid taking public transportations.	97.9
K12. People who have contact with someone infected with the COVID-19 virus should be immediately isolated in a proper place. In general, the observation period is 14 days.	95.1
K7. The COVID-19 virus spreads via respiratory droplets of infected individuals.	92.1
K1. The main clinical symptoms of COVID-19 are fever, fatigue, dry cough, and myalgia.	91.5
K3. There currently is no effective cure for COVID-2019, but early symptomatic and supportive treatment can help most patients recover from the infection.	89.3
K4. Not all persons with COVID-2019 will develop to severe cases. Only those who are elderly, have chronic illnesses, and are obese are more likely to be severe cases.	69.5
K2. Unlike the common cold, stuffy nose, runny nose, and sneezing are less common in persons infected with the COVID-19 virus.	68.7
K8. Ordinary participants can wear general medical masks to prevent the infection by the COVID-19 virus.	57.0
K5. Eating or contacting wild animals would result in the infection by the COVID-19 virus.	30.3
K6. Persons with COVID-2019 cannot infect the virus to others when a fever is not present.	9.2
K9. It is not necessary for children and young adults to take measures to prevent the infection by the COVID-19 virus.	4.1

Note: Questionnaire adapted from Zhong BL, Luo W, Li HM, et al. Knowledge, attitudes, and practices towards COVID-19 among Chinese residents during the rapid rise period of the COVID-19 outbreak: a quick online cross-sectional survey. Int J Biol Sci. 2020;16(10):1745-1752. Published 2020 Mar 15. doi:10.7150/ijbs.45221.

**Table 4:** COVID-19 knowledge score by participants’ characteristics (*n* = 1208).

	COVID-19 Knowledge Score		
Variable	Mean	SD	*p*-Value
Country			
Jordan	8.44	1.46	0.000 ***
Saudi Arabia	7.79	1.57	
Kuwait	7.59	1.99	
**Gender**			
Males	7.86	1.94	0.263
Females	7.97	1.61	
**Age**			
18–29 years	7.72	2.04	0.006 **
30–49 years	8.01	1.49	
50 years and above	8.11	1.55	
**Marital status**			
Single	7.75	2.07	0.002 **
Married	8.07	1.46	
Divorced	7.47	1.72	
Widowed	8.19	1.56	
**Education level**			
Completed secondary grade or lower	7.72	2.01	0.015 *
Completed bachelor degree	7.94	1.70	
Completed higher education	8.18	1.31	
**Employment status**			
Retired	7.82	2.02	0.092
Unemployed	7.97	1.78	
Employed	8.04	1.51	
University students	7.70	1.91	
**Income**			
$705 or lower	8.01	1.70	0.094
$705–1408	8.24	1.69	
$1408–2113	7.81	1.65	
$2113 and above	7.92	1.38	
**In recent days, have you gone to any crowded place?**			
Yes	8.11	1.89	0.325
No	7.99	1.49	
**In recent days, have you worn a mask when leaving home?**			
Yes	8.16	1.51	0.001 **
No	7.86	1.59	

* *p* < 0.05, ** *p* < 0.01, *** *p* < 0.001.

**Table 5:** Multiple regression analysis predicting participants’ COVID-19 knowledge.

	Model 1 ^a^		
Variable	B	SE	ß
Demographic data			
Country			
Saudi Arabia	−0.804	0.124	−0.247 ***
Kuwait	−1.005	0.148	−0.302 ***
Age			
30–49 years	0.285	0.151	0.090
50 years and above	0.460	0.193	0.125 *
Gender			
Females	0.045	0.101	0.014
Marital status			
Married	0.008	0.135	0.003
Divorced	−0.588	0.241	−0.082 *
Widowed	−0.041	0.317	−0.004
Educational level			
Completed bachelor degree	0.106	0.125	0.033
Completed higher education	0.226	0.163	0.055
Employment status			
Unemployed	−0.225	0.203	−0.057
Employed	−0.139	0.176	−0.044
University students	−0.566	0.236	−0.134 *
Income			
$705–1408	−0.027	0.148	−0.006
$1408–2113	−0.163	0.213	−0.026
$2113 and above	−0.065	0.164	−0.021
Constant			8.486
Adjusted R^2^			0.073
*p*-value			*p* < 0.001

* *p* < 0.05, *** *p* < 0.001. a: includes country, age, gender, marital status, educational level, employment status, and income level. B: the average change in the dependent variable associated with a one unit change in the independent variable, statistically controlling for the other independent variables; SE: the standard deviation of its sampling distribution or an estimate of that standard deviation; ß: a statistical measure that compares the strength of the effect of each independent variable on the dependent variable.

## Data Availability

The data presented in this study are available on request from the corresponding author.
