# Correction: Naser et al. Knowledge and Practices during the COVID-19 Outbreak in the Middle East: A Cross-Sectional Study. *Int. J. Environ. Res. Public Health* 2021, *18*, 4699

**DOI:** 10.3390/ijerph19063466

**Published:** 2022-03-15

**Authors:** Abdallah Y. Naser, Eman Zmaily Dahmash, Zahra Khalil Alsairafi, Hassan Alwafi, Hamad Alyami, Zahraa Jalal, Ahmed M. Al Rajeh, Vibhu Paudyal, Yosra J. Alhartani, Fawaz Mohammad Turkistani, Fadi Fouad Hassanin

**Affiliations:** 1 Department of Applied Pharmaceutical Sciences and Clinical Pharmacy, Isra University, Amman 11622, Jordan; eman.zmaily@iu.edu.jo (E.Z.D.); yosra.alharatni@hotmail.com (Y.J.A.); 2 Department of Pharmacy Practice, Kuwait University, Kuwait City 12037, Kuwait; zahra.alsairafi@hsc.edu.kw; 3 Department of Pharmacology and Toxicology, Umm al-Qura University, Mecca 21514, Saudi Arabia; hassan.alwafi.16@ucl.ac.uk; 4 Department of Pharmaceutics, Najran University, Najran 1988, Saudi Arabia; hsalmukalas@nu.edu.sa; 5 School of Pharmacy, University of Birmingham, Edgbaston, Birmingham B15 2TT, UK; z.jalal@bham.ac.uk (Z.J.); v.paudyal@bham.ac.uk (V.P.); 6 Department of Respiratory Care, King Faisal University, Al Hofuf 31982, Saudi Arabia; amalrajeh@kfu.edu.sa; 7 Alnoor Hospital, Mecca 21514, Saudi Arabia; fm_turkistani@hotmail.com; 8 Ophthalmology Department, College of Medicine, Jeddah University, Jeddah 2749, Saudi Arabia; ffhassanin@gmail.com

The authors would like to make the following corrections about the published paper [1].

**Replacing the Data Availability Statement from:** Not applicable.

**Data Availability Statement:** The data presented in this study are available on request from the corresponding author.
